# Vestibular Schwannoma: What We Know and Where We are Heading

**DOI:** 10.1007/s12105-020-01155-x

**Published:** 2020-03-30

**Authors:** Vinay Kumar Gupta, Arjuna Thakker, Keshav Kumar Gupta

**Affiliations:** 1 College of Medical and Dental Sciences, University of Birmingham, Birmingham, B15 2TT UK; 2 Royal Wolverhampton NHS Trust, Wolverhampton, WV10 0QP UK

**Keywords:** Vestibular, Schwannoma, Review, Pathology, Diagnosis, Management

## Abstract

Vestibular schwannoma (VS) is a Schwann cell-derived tumour arising from the vestibulocochlear nerve. Although benign, it represents a threat to intracranial structures due to mass effect and carries a small risk of malignant transformation. VS therefore represents an important healthcare burden. We review the literature regarding pathogenesis, risk factors, and diagnosis of VS. The current and future potential management strategies are also discussed. A narrative review of all relevant papers known to the authors was conducted. The majority of VS remain clinically stable and do not require interventional procedures. Nevertheless, various surgical techniques exist for removing VS, the most common of which are translabyrinthine and retrosigmoid approaches. Due to surgical risks such as hearing loss, facial nerve dysfunction, post-operative headache, and cerebrospinal fluid leakage, a "watch and rescan" approach is adopted for most patients. Radiotherapy is a useful alternative and has been shown to have a similar response for growth restriction. Due to the heterogeneous nature of VS, there is a lack of consensus regarding management of tumours that are too large for conservative management but too small to indicate surgery. Emerging biologic therapies, such as Bevacizumab, Everolimus, and Lapatinib, as well as anti-inflammatories like aspirin are promising potential treatments; however, long-term evidence of their efficacy is required. The knowledge base regarding VS continues to improve. With increased understanding of the pathogenesis of these tumors, we believe future work should focus on pharmacologic intervention. Biologic therapies aimed toward improved patient outcomes are particularly promising.

## Introduction

Vestibular schwannoma (VS), also called acoustic neuroma, is a benign Schwann cell-derived tumour arising from the vestibulocochlear nerve. These tumors represent 85% of intracranial growths arising at the cerebellopontine angle [1]. The Koos grading scale is commonly used to classify tumour size with respect to extrameatal extension and brainstem compression (Table 1) [2]. There is an ongoing debate as to the terminology of VS versus acoustic neuroma. As the majority of tumours arise from the vestibular aspect of the vestibulocochlear nerve and the tumour cells are Schwann cells rather than neuronal, we prefer “vestibular schwannoma” for this article [3].

**Table 1:** Koos grading system for vestibular schwannoma

Koos grade	Description
I	Intracanalicular
II	Extension into cerebellopontine angle, < 2 cm
III	Occupies cerebellopontine angle, no brainstem displacement, < 3 cm
IV	Brainstem displacement, > 3 cm

Although benign, VS represents a risk to various intracranial structures due to mass effect. The most common symptoms include progressive hearing loss and tinnitus which are reported in over 60% of patients. Larger tumours can cause hydrocephalus and brainstem compression leading to symptoms such as facial paraesthesia, vertigo, and headache [4].

VS accounts for approximately 8% of all intracranial tumours with an incidence of 10.4 per million per year [5]. The majority of tumors are unilateral and sporadic. Bilateral disease accounts for less than 5% of cases and is a hallmark of a hereditary disease related to neurofibromatosis type 2 (NF2). Patients typically present between the ages of 20–40; however, those associated with NF2 often manifest earlier [6, 7]. The documented incidence of VS is rising, but there is a general consensus that this is a reflection of increased reporting. The now common use of magnetic resonance imaging (MRI) for symptoms of tinnitus and earlier care seeking patient behaviours are contributing factors to these higher numbers [8].

This article aims to provide an up to date review of our understanding of the pathogenesis and diagnosis of VS with a focus on current management strategies. Emerging treatment options are also discussed and explored.

## Histologic Features

**Fig. 1:**
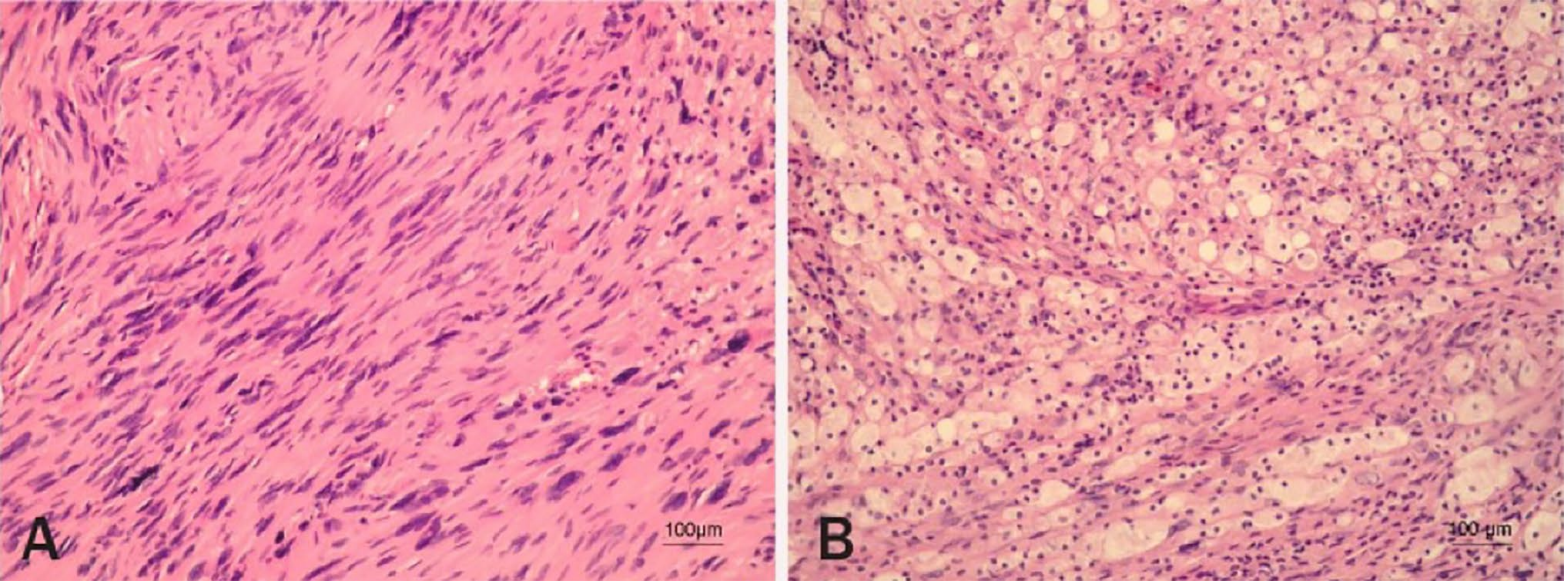
Illustration of a histologic sample of vestibular schwannoma. **a** Antoni A tissue. Areas of tumor containing abundant spindle cells, with twisted nuclei and indistinctive cytoplasmic borders, arranged in short, well-composed bundles. Acellular pathognomonic features of a schwannoma, called Verocay bodies, are also seen. **b** Antoni B tissue. Areas of tumor composed of loosely arranged Schwann cells laden with foamy macrophages and surrounding foci of necrosis, cystic changes, and haemorrhage. In some tumor cells, degenerative nuclear changes can be seen (200 × , hematoxylin and eosin). Reproduced with permission from ‘’Frequency of loss of heterozygosity of the NF2 gene in schwannomas from Croatian patients’’. Reproduced with permission from Pećina-Slaus et al. [10]

The majority of VS grow from the inferior vestibular nerve with rare cases arising from the superior vestibular, or cochlear portion of the nerve [9]. The histologic features of bipolar spindle cells arranged in distinctive Antoni A and Antoni B tissue types are characteristic (Fig. 1).

## Pathogenesis

### Molecular Pathogenesis

**Fig. 2:**
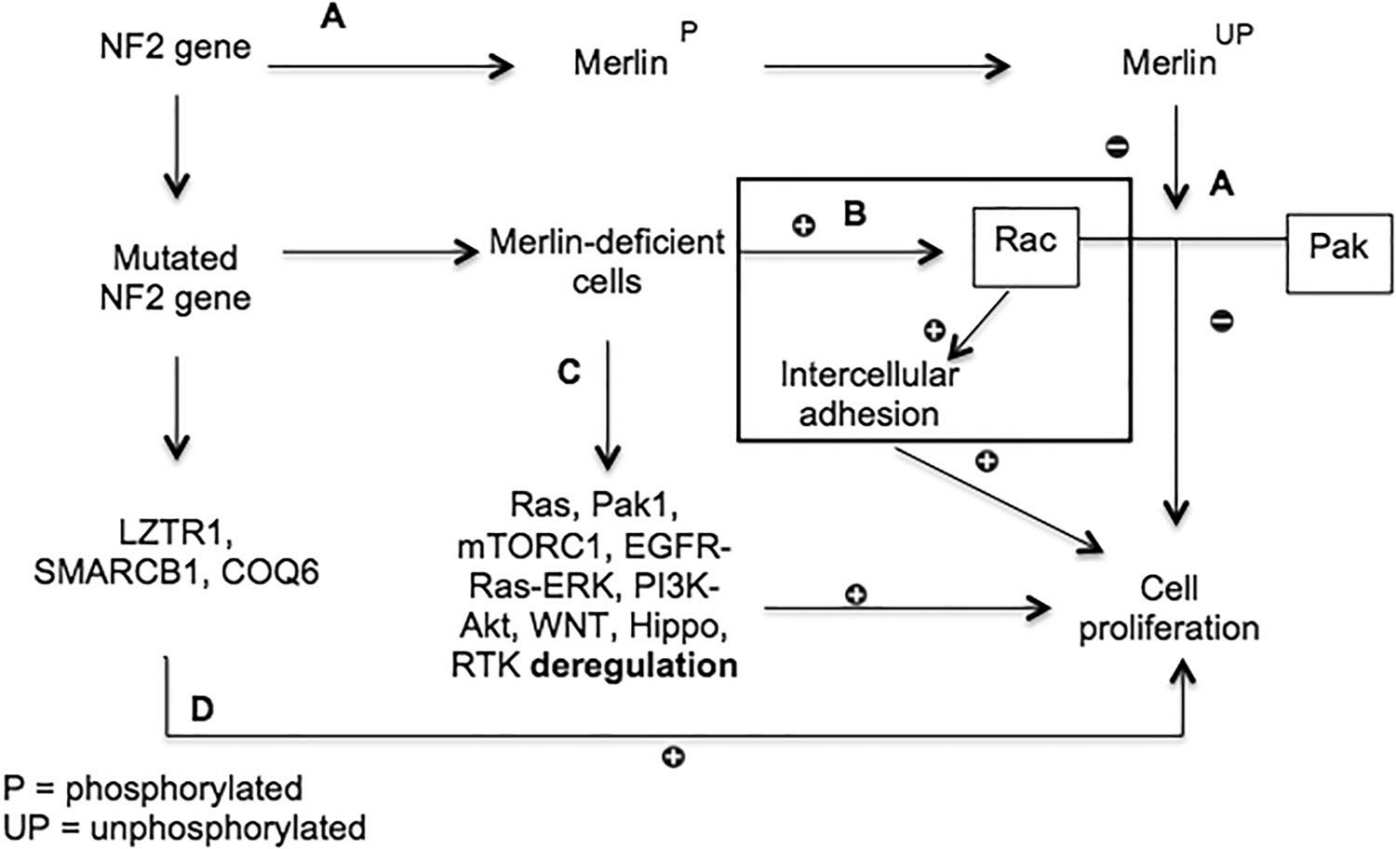
Mechanisms of NF2 gene-related VS development. (A) In a steady-state, unphosphorylated merlin restricts cell proliferation by inhibiting Rac and p21-activated kinase (Pak) [14]. (B) NF2 gene mutations lead to merlin deficient cells, causing Rac activation leading to intercellular adhesion and cell proliferation [15]. (C) Merlin deficient cells also deregulate various intracellular pathways causing cell proliferation [12]. (D) Mutations to *NF2* also affect other pathways, seen in schwannomatosis, leading to eventual cell proliferation—although this is poorly understood [16]

Mutations to *NF2*, a tumour suppressor gene on chromosome 22, play a vital role in the development of both sporadic and NF2-related disease [11]. Inactivation of the *NF2* protein product, Merlin (schwannomin), leads to deregulation of various intracellular signalling pathways such as Rac1, Ras, PAK1, and mTORC1. Inactivation of other tumour suppressor genes including *LZTR1*, *SMARCB1*, and *COQ6* are also linked to schwannoma development (Fig. 2) [12, 13].

Although the role of *NF2* mutations was reinforced by recent large-scale sequencing studies, there are data to suggest that NF2-associated VS has a different, polyclonal mutation pattern [17]. This has been postulated to account for variance in treatment outcomes as compared to sporadic VS [18]**.**

### Risk Factors

Presently, much of the research on risk factors for development of VS is focused on radiation exposure and mobile phone use. High dose ionizing radiation exposure (mean dose 4.6 ± 1.9 Gy) to the cerbellopontine angle in children has been linked to a higher incidence of VS after a latency period of 20–55 years [19]. Other studies from atomic bomb survivors in Japan revealed similar trends [20, 21]. The contrary has been observed with lower dose radiation where no significant link was found between the use of ionizing medical imaging and VS development (odds ratio (OR) 0.97, 95% confidence interval (CI) 0.54–1.75) [22].

Multiple epidemiological studies failed to establish a significant link between mobile phone exposure and VS [23–25]. However, a 2009 systematic review and meta-analysis evaluating long-term mobile phone use found a 60% increase in risk for VS following ten years of ipsilateral mobile phone usage (OR 1.6, 95% CI 1.1–2.4) [26]. These findings led to a call for awareness of governments to this risk and even for the classification of mobile phone radiation as a probable human carcinogen [27, 28]. A potential flaw to these data is that most studies relied on patient recall of mobile phone usage introducing recall bias. Furthermore, the majority of the data relied on MRI to confirm the diagnosis without the analysis of tumour histopathology leaving the potential for inaccuracies in the reported findings. Other potential risk factors studied include smoking, occupation, allergic rhinitis, noise exposure, asthma, and eczema [29].

### Diagnosis

VS is often diagnosed due to otological or neurological symptoms. Otological symptoms include progressive sensorineural hearing loss, unilateral tinnitus, and vertigo. These are more common than the neurological symptoms such as trigeminal and facial nerve impairment, headache, and hydrocephalus amongst the general population [4, 30].

Up to 20% of patients presenting to Ear, Nose, and Throat (ENT) clinics have symptoms suggesting a lesion at the cerebellopontine angle [31]. Therefore, patients presenting with the aforementioned symptoms are generally investigated with otoscopy, pure tone audiometry, and MRI of the internal acoustic meatus. As hearing loss typically presents first, the use of brainstem-evoked response audiometry has proven to be a useful screening tool for a suspected diagnosis. Despite this, it is no longer used as a first-line investigation due to high false-negatives, up to 30%, for small schwannomas and a false positive rate of 10% [32].

All patients presenting with unilateral tinnitus or sensorineural hearing loss require MRI as the gold standard investigation [30, 33]. Fortnum et al. conducted a systematic review and cost-effectiveness study on the use of MRI in diagnosis of VS. Although the use of gadolinium-enhanced T1 weighted MRI is considered the gold standard, there was little difference in sensitivity and specificity compared to non-contrast T2 weighted scans. Furthermore, the use of non-contrast T2 weighted scans was deemed more cost-effective for clinical practice [34].

### Current Treatment Options

Various approaches exist to treat patients with VS. These include observation, termed a ‘watch and rescan’ approach, surgical removal, and radiotherapy. The main aim of interventional treatment is to remove or debulk the tumour to prevent mass effect [30].

### Conservative

Observation is an accepted treatment option due to the slow growth rate of VS. A systematic review of 41 papers showed a mean tumour growth rate of 1–2 mm/year with up to 75% of tumours showing no further growth [35]. An observational study of 436 patients showed similar findings where 68% of tumours did not grow during the follow-up period (mean 3.6 years). A third study demonstrated that patients with NF2-related disease had higher mean growth rates (1.7 mm/year) as compared to sporadic tumours (1.1 mm/year) [36]. Interestingly, one study observed that sporadic tumours reaching two centimetres in intracranial diameter are likely to continue growing. Despite this, there is no accepted cut-off to dictate when an observational approach transitions to an interventional one. Other cited risk factors for increased growth rates include the presence of an extracanalicular component and diagnosis at a young age; however, these observations are based on limited sample sizes from their respective studies [30, 37].

Although observation can increase the risk of tumour progression and mass effect, it seems a safe approach due to the minimal growth rate. Furthermore, delaying surgical intervention appears to have no increased risk in mortality [38]. Since growth usually manifests within the first 3 years after presentation, a recommended protocol for observation is serial MRI scans at 6 month intervals for 2 years and then another scan 2 years later. Following this, patients should be scanned every 5 years with lifelong follow-up [39].

### Surgery

A range of surgical methods for VS removal are discussed in the literature and mainly pertain to large tumours. The approaches include: middle fossa (MF), extended middle fossa, transotic, and endoscopic resection. The translabyrinthine (TL) and retrosigmoid (RS) approaches are the most commonly used in the United Kingdom (UK) and are further discussed below [30]. The benefit of the MF approach for small tumours of greater hearing preservation versus the RS approach is worth mentioning. However, its use is now limited in the UK due to high risks of damage to the facial nerve and seizures caused by temporal lobe manipulation [40, 41].

The TL approach refers to a retroauricular incision with posterior extension toward the mastoid tip. This provides quick access to the internal acoustic canal, facial nerve, and tumour. A major disadvantage is poor preservation of hearing, limiting the use of the TL approach to those with minimal or absent preoperative hearing.

With the RS approach, a suboccipital incision exposes the transverse and sigmoidal sinuses, and subsequent cerebellar manipulation exposes the internal acoustic canal. Although this method is superior in preserving hearing, there is an increased risk of facial and cochlear nerve damage [30]. Of course, surgical intervention within the cranium is not without other risks such as meningitis, cerebellar injury, epilepsy, persistent headache, and cerebrospinal fluid leakage [42–45].

In a systematic review of 35 studies (n = 5064), it was reported that facial nerve dysfunction was less likely to occur with the RS versus TL approach for tumours greater than 1.5 cm [40]. Evidence exists to suggest the size of the tumour preoperatively is an important prognostic factor for facial nerve dysfunction postoperatively [46]. The study compared surgical interventions based on tumour size which represents a potential confounder to the data. They also reported postoperative headaches and CSF leak rate to be significantly greater following the RS approach versus TL. It is postulated that postoperative headaches are more common with the RS approach due to the incision size and remnant intracranial bone dust [30]. Conflicting data exists regarding CSF leak; Magnus et al. reported no difference between RS and TL approaches in 1,922 patients while Sughrue et al. found the TL approach was riskier [47, 48].

Mortality and tumour recurrence did not differ between surgical approaches with mortality rates as low as 0.2% [48]. Non-specific neurological complications such as cerebellar dysfunction, stroke, and epilepsy were rare and occurred in less than 3% of cases [40]. The impact of surgery on patient well-being was notable as the procedure potentially delays a return to work for up to four months. This may lead to financial hardship and adverse affects on mental health [49].

### Radiotherapy

There are three forms of radiotherapy treatment for VS management: stereotactic radiosurgery (SRS), fractionated stereotactic radiotherapy (FSRT), and proton beam therapy. SRS and FRST are most commonly used, as there is limited availability of proton beam therapy and inadequate evidence of its efficacy [50]. The main aim of radiotherapy is to prevent tumour growth, therefore this is not considered a suitable approach for large tumours with mass effect.

Various advantages and disadvantages apply to SRS and FRST. SRS uses Gamma Knife technology to expose the tumour to a singular dose of radiation and is less applicable to large lesions (> 2.5 cm extracanalicular diameter) [51]. FRST requires numerous sessions of radiotherapy in an attempt to target the tumour at the most radiation-sensitive phase of the cell cycle for, theoretically, greater efficacy [52]. In addition, FRST systems are more readily available in hospitals and can be used on larger lesions [51].

A recent systematic review comparing FRST and SRS reported similar rates of tumour control with an average of 4.8% and 5% of patients undergoing treatment requiring rescue therapy, respectively. Facial and trigeminal nerve deterioration was less for SRS. These comparisons were based on limited evidence; however, and without randomised controlled trials (RCTs). Furthermore, only two studies on FRST were evaluated limiting the validity of estimates regarding this treatment. More studies are required to confidently compare these two therapies [53].

Ranges of controlled studies demonstrate comparable progression-free survival rates and side effects, such as nerve palsies and hearing deterioration, between radiotherapy and microsurgery [54–61]. A recent Cochrane review highlighted that these comparisons are based on low-quality evidence, and no RCTs exist that compare surgery and radiotherapy treatments [62]. Additionally, long term evidence (> 10 years) regarding hearing preservation following radiotherapy is limited. Yang et al. reported an average hearing preservation rate of 57% from data derived from 74 articles; however, the average follow-up was only 41.2 months [63]. A more recent case-controlled study found that hearing preservation amongst patients decreases from 53% at 5 years to 34% at 10 and 15 years amongst all tumour grades [64]. This emerging evidence at increased time points suggests hearing may not be as well preserved as once thought, and loss occurs due to longer-term nerve damage as a result of radiation exposure. These studies also found the tumour Koos grade to be an independent predictor of hearing loss, representing a possible confounder.

The evidence presented is inconclusive regarding the best treatment options for all categories of VS. While most small tumours are managed conservatively and larger tumours with surgery and/or radiotherapy, there is a grey area surrounding best management options for tumours falling between categories [65]. More robust, high quality RCTs are required to guide treatment in these scenarios.

### Emerging Treatments

As knowledge surrounding the molecular pathology of VS improves, targeted biologic therapies are emerging at the forefront of treatment. Bevacizumab, Everolimus, and Lapatinib are potential options for treating VS. Furthermore, a link between tumour growth and non-steroidal anti-inflammatory medications such as aspirin has been recognised.

Bevacizumab is a monoclonal antibody and vascular endothelial growth factor (VEGF) inhibitor. VEGF is a key mediator of angiogenesis and subsequently aids tumour growth [66]. Plotkin et al. conducted the earliest research on the use of Bevacizumab in NF2 patients with progressive disease. Although these studies reported improvement in hearing and restriction of tumour growth in over 50% of patients, they were based on limited sample sizes (n = 10 and n = 31, respectively). The later 2012 study was also based on retrospective data with a median treatment duration of only 14 months [67, 68].

Since then, more promising data has arisen. In a systematic review on the safety and efficacy of Bevacizumab, 41% of patients receiving treatment had tumour regression, 20% experienced hearing improvement, and 69% had no further hearing deterioration [69]. This promising evidence supports the funding of Bevacizumab by NHS England for VS treatment; however, these data were only based on NF2 patients. Therefore is difficult to know whether the results will translate to the majority of the sporadic VS cases [70, 71].

Furthermore, various side effects have been reported in a dose-dependent relationship with Bevacizumab including hypertension, proteinuria, and infection [69, 72]. Although the most recent systematic review by the Congress of National Neurological Surgeons (CNS) supports the use of Bevacizumab, the recommendations are based on level 3 evidence [73]. It seems clear more long-term evidence pertaining to safety and appropriate dosing is required.

Everolimus is an mTOR complex 1 (mTORC1) inhibitor. mTORC1 activation has been implicated in tumour growth as a result of merlin deficiency. Furthermore, inhibition of mTORC1 has demonstrated antiangiogenic properties [74, 75]. In theory, this seems a promising treatment; however, there is limited evidence of its clinical use. Phase II trials in NF2 patients have shown mixed results. Karajannis et al. found no response to Everolimus on tumour growth or hearing improvements [76]. Although Goutagny et al. found a 66.5% reduction in tumour growth during Everolimus use in ten NF2 patients, growth resumed following the discontinuation of treatment [77]. At the time of writing, the CNS does not support use of Everolimus for VS management [73].

Lapatinib is an EGFR/ErbB2 inhibitor with demonstrated promise for tumor growth inhibition during in vitro trials [78]. The CNS recognises Lapatinib as a potential agent for growth management and hearing improvement due to encouraging early clinical evidence [73]. A phase II clinical trial involving 21 NF2 patients found a ≥ 15% decrease in tumour volume in 23.5% of participants following serial MRI scans, and 30.8% of participants showed improved hearing. Only 14 of the 21 participants were eligible for evaluation of audiological response, however. The lack of a control group also limits these findings. Encouragingly, there were low levels of reported toxicity with Lapatinib which may prove important when comparing to Bevacizumab and its propensity for adverse side effects [79]. Again, long-term controlled studies are required to provide more robust evidence.

Studies also suggest that aspirin may be beneficial to delay tumour growth through its anti-cyclooxygenase 2 (COX-2) effect. A proliferative effect of COX-2 expression in 30 tumours was observed [80]. Another study demonstrated the application of acetylsalicylic acid decreased COX-2 expression and in vitro cell growth [81]. Furthermore, a retrospective analysis followed 347 patients with VS, taking aspirin for unrelated reasons, to inspect the effect on volumetric growth versus non-aspirin users. They found a significant inverse association with aspirin users and tumour growth (OR 0.32, 95% CI 0.11–0.91) [82]. Although two similar retrospective analyses did not find an inverse association, they did not observe increased tumour growth with aspirin or other NSAID users [83, 84]. Therefore, a level 3 recommendation exists in the CNS stating aspirin may be considered for patients undergoing observation [73].

More recent evidence is not as promising. In a 2019 study, tissue samples of 1048 tumours were analysed. It was discovered that while COX-2 expression increased with tumour proliferation, the use of acetylsalicylic acid did not alter COX-2 expression [85]. This study used tumour tissue retrospectively which can lend itself to selection bias since large tumours are more likely to be managed surgically. Most of the tissue samples were likely obtained from clinically stable tumours that may not have grown independent of the presence of the acetylsalicylic acid. In any case, the conflicting research on the use of aspirin necessitates further investigated through large-scale trials. Currently, there is one prospective phase II randomised double blind trial recruiting patients to assess the effect of acetylsalicylic acid on tumour growth and hearing preservation (NCT03079999).

## Conclusions

VS is a clinically important disease with an evolving knowledge base. There is now a vast scope of literature pertaining to risk factors, diagnosis, and treatment. With improved understanding of the pathogenesis, future work should focus on biologic interventions due to the current risks and lack of high quality RCTs of different surgical and radiologic treatment options.
